# Supplementary material for: Screening of In-Vitro Anti-Inflammatory and Antioxidant Activity of Sargassum ilicifolium Crude Lipid Extracts from Different Coastal Areas in Indonesia
Source: Mar Drugs. 2021 Apr 28;19(5):252. doi: 10.3390/md19050252 (PMC8146618; doi:10.3390/md19050252)
Supplement: Supplementary file 1 [file marinedrugs-19-00252-s001.zip › marinedrugs-1175723-supplementary.pdf]

Supplementary Materials for the Article

Screening of In-vitro Anti-Inflammatory and Antioxidant Activity of *Sargassum cristaefolium* Crude Lipid Extracts from Different Coastal Areas in Indonesia

Saraswati [1], Puspo Edi Giriwono [1,2], Diah Iskandriati [3], Nuri Andarwulan [1,2*]

[1] Department of Food Science and Technology, Faculty of Agricultural Engineering and Technology, IPB University (Bogor Agricultural University), West Java, Indonesia 16680
[2] Southeast Asian Food and Agricultural Science Technology (SEAFAST) Center, IPB University (Bogor Agricultural University), West Java, Indonesia 16680
[3] Primate Research Center, IPB University (Bogor Agricultural University), West Java, Indonesia 16151
*Correspondence: andarwulan@apps.ipb.ac.id; Tel: ++62-251 8626725[2]

List of Figures

Figure S1: GC-FID chromatogram of FAME (fatty acid methy ester) mix C4-24 (external standard) according to study of Saraswati *et al.* [38] 2
Figure S2: GC-FID chromatogram of SHB's FAME according to study of Saraswati *et al.* [38] 3
Figure S3: GC-FID chromatogram of UGB's FAME according to study of Saraswati *et al.* [38] 4
Figure S4: GC-FID chromatogram of PI's FAME according to study of Saraswati *et al.* [38] 5
Figure S5: GC-FID chromatogram of AB's FAME according to study of Saraswati *et al.* [38] 6

List of Tables

Table S1: Selectivity index (SI) of UGB and AB treatment on RAW 264.7 cells 7
Table S2: The results of Pearson's correlation analysis between lipid-soluble compounds and bioactivities (anti-inflammatory and antioxidant) 8

Figure S1 GC-FID chromatogram of FAME (fatty acid methy ester) mix C4-24 (external standard) according to study of Saraswati *et al.* [38]

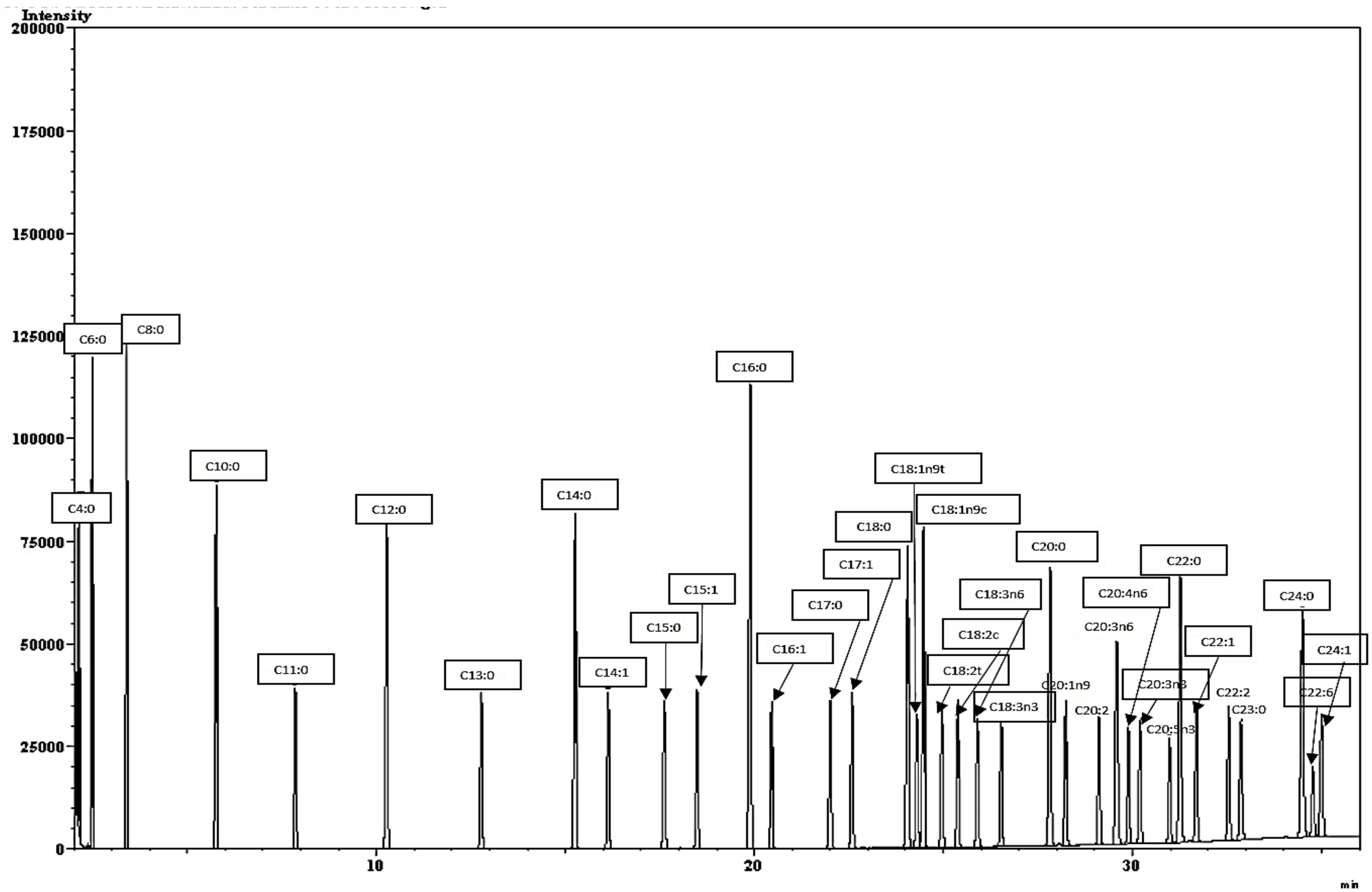

Figure S2 GC-FID chromatogram of SHB's FAME according to study of Saraswati *et al.* [38]

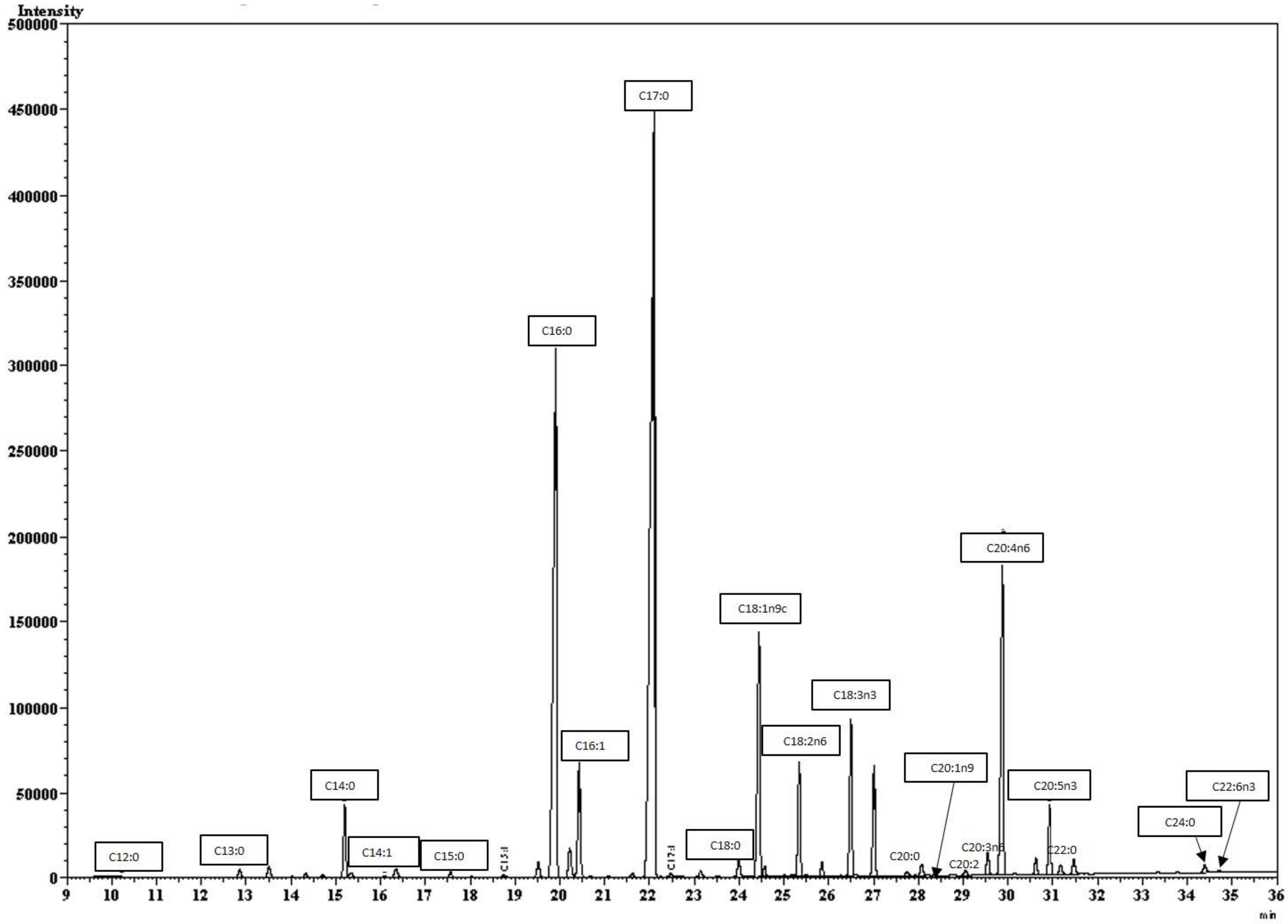

Figure S3 GC-FID chromatogram of UGB's FAME according to study of Saraswati *et al.* [38]

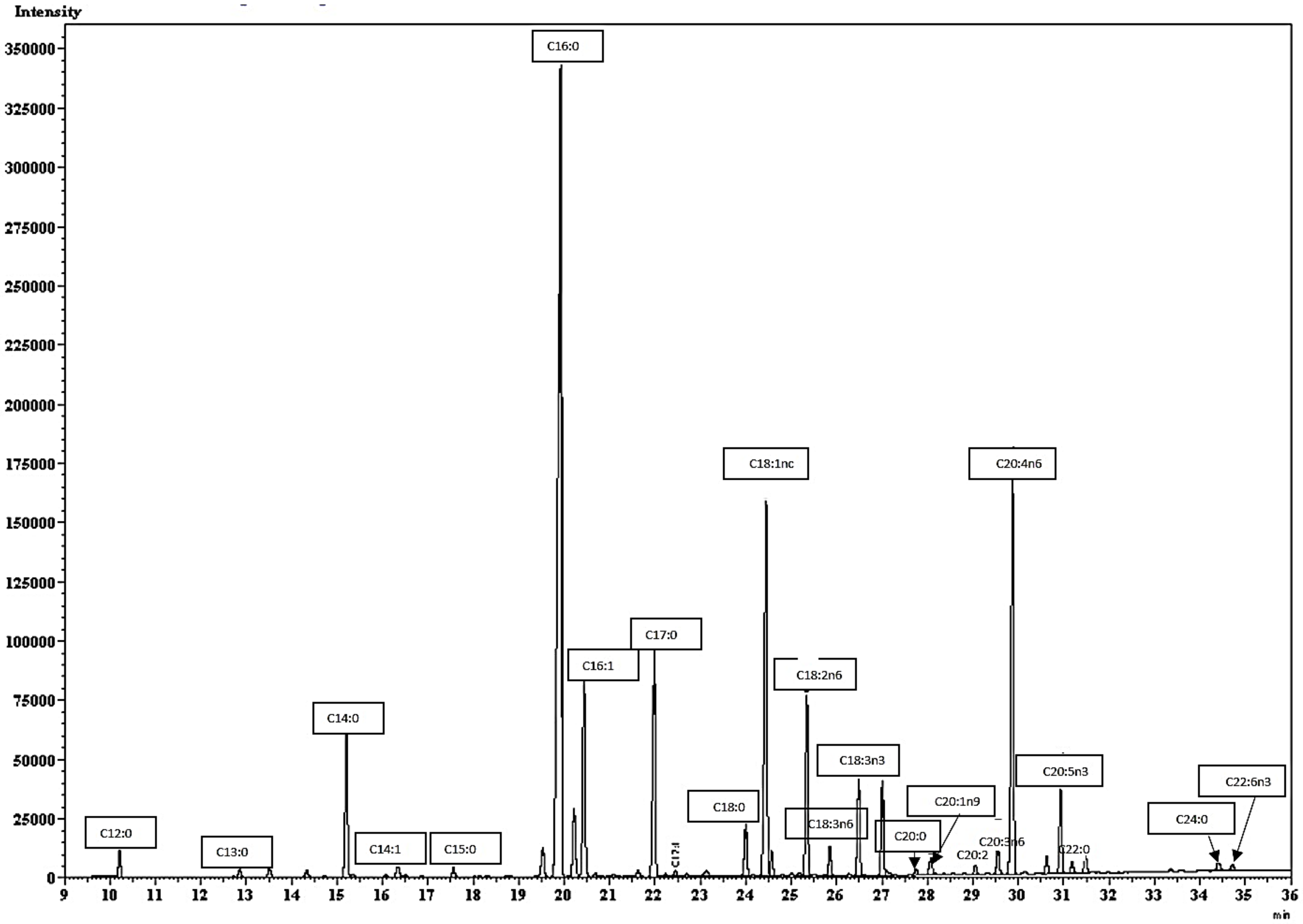

Figure S4 GC-FID chromatogram of PI's FAME according to study of Saraswati *et al.* [38]

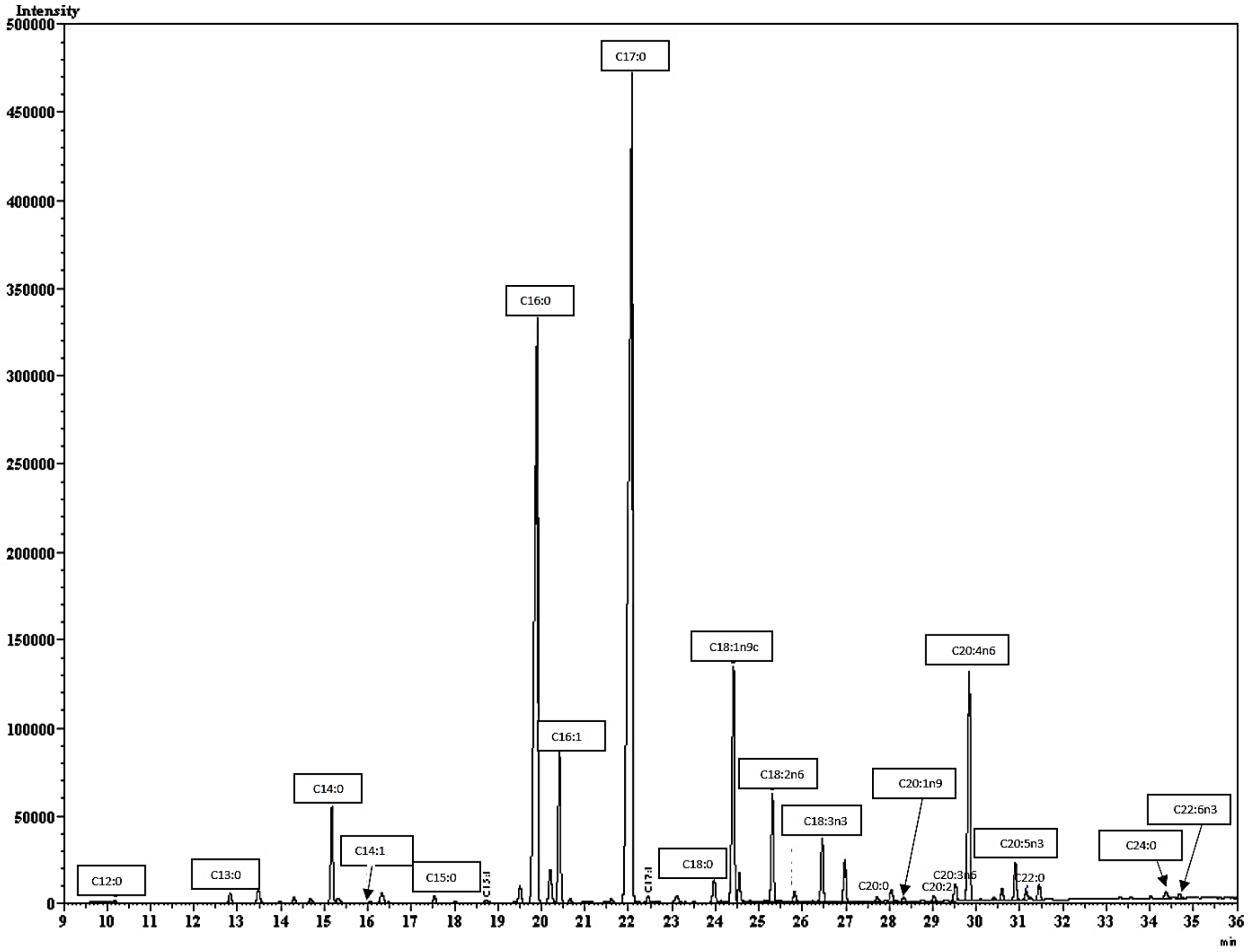

*Note: Internal standard (heptadecanoic acid) added at the time of methylation of SHB and PI samples was 10 times more concentrated than those of UGB and AB samples

Figure S5 GC-FID chromatogram of AB’s FAME according to study of Saraswati *et al.* [38]

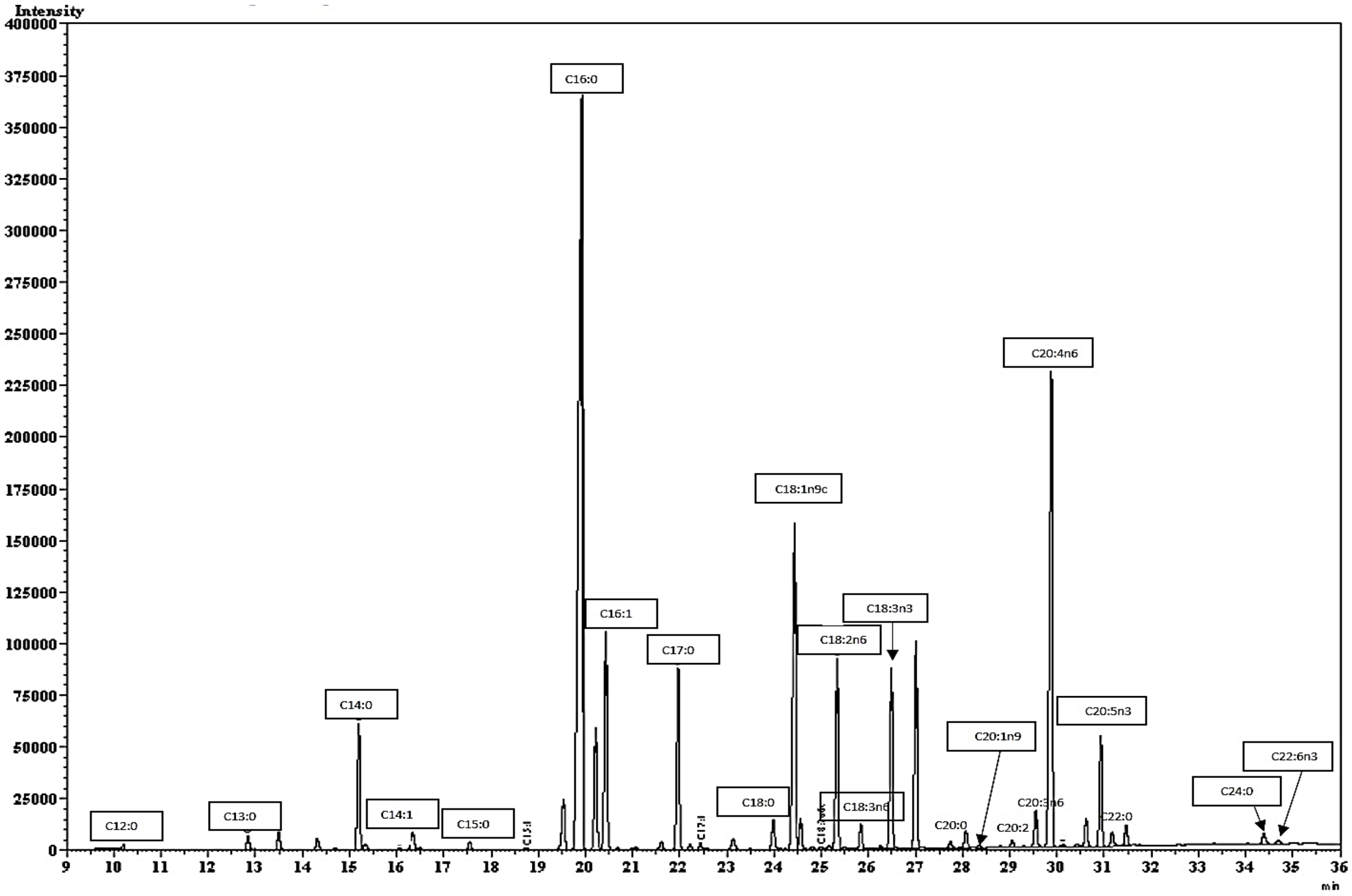

*Note: Internal standard (heptadecanoic acid) added at the time of methylation of SHB and PI samples was 10 times more concentrated than those of UGB and AB samples

Table S1 Selectivity index (SI) of UGB and AB treatment on RAW 264.7 cells

Sample	CC50 (μg/ml)	IC50 (μg/ml)	Selectivity Index (CC50/IC50)
**Pre-incubated cell culture model**			
UGB	55.15	25.44	2.17
AB	120.8	37.81	3.19
**Co-incubated cell culture model**			
UGB	55.15	35.76	1.54
AB	120.8	59.62	2.03

Note: CC50 is the half maximal cytotoxic concentration, IC50 is the half maximal LPS-induced NO inhibitory concentration.

Table S2 The results of Pearson's correlation analysis between lipid-soluble compounds and bioactivities (anti-inflammatory and antioxidant)

Lipid-soluble compounds	DPPH scavenging activity		FRAP		NO-inhibition in pre-incubated cell culture model (at 25 μg/ml)		NO-inhibition in co-incubated cell culture model (at 25 μg/ml)		NO-inhibition in pre-incubated cell culture model (at 50 μg/ml)		NO-inhibition in co-incubated cell culture model (at 50 μg/ml)	
	Pearson Correlation	Sig. (2-tailed)	Pearson Correlation	Sig. (2-tailed)	Pearson Correlation	Sig. (2-tailed)	Pearson Correlation	Sig. (2-tailed)	Pearson Correlation	Sig. (2-tailed)	Pearson Correlation	Sig. (2-tailed)
Neutral lipid	.764**	.004	.121	.709	.413	.182	.753**	.007	.577*	.050	.483	.111
Glycolipid	-.440	.152	-.129	.689	.018	.956	-.425	.193	-.422	.171	-.331	.293
Phospholipid	-.233	.465	.020	.951	-.369	.238	-.231	.494	-.090	.780	-.098	.763
Chlorophyll a	.527	.078	.031	.924	.472	.122	.668*	.025	.275	.387	.300	.343
Chlorophyll c	.139	.666	-.164	.611	.263	.409	.277	.409	-.034	.915	-.029	.929
Fucoxanthin	-.172	.592	-.469	.124	-.085	.793	-.060	.860	-.380	.223	-.390	.211
Carotene	-.869**	.000	-.686*	.014	-.852**	.000	-.972**	.000	-.905**	.000	-.918**	.000
Total fatty acid	-.096	.766	.220	.492	.406	.191	.008	.981	.142	.659	.107	.740
C12:0	.452	.140	.707*	.010	.651*	.022	.558	.075	.565	.056	.643*	.024
C14:0	.756**	.004	.401	.196	.371	.235	.746**	.008	.665*	.018	.701*	.011
C14:1	.304	.337	.176	.584	.553	.062	.479	.136	.220	.491	.258	.418
C15:0	.474	.120	-.007	.984	-.114	.723	.401	.221	.288	.365	.293	.356
C16:0	.682*	.015	.107	.742	.145	.652	.683*	.020	.476	.118	.511	.089
C16:1	.846**	.001	.190	.553	.351	.263	.836**	.001	.607*	.036	.629*	.029
C17:1	.545	.067	-.076	.814	-.092	.777	.501	.116	.327	.299	.314	.319
C18:0	.676*	.016	.708**	.010	.610*	.035	.714*	.014	.735**	.006	.785**	.002
C18:1n9c	.235	.461	.577*	.050	.200	.534	.151	.657	.411	.185	.447	.145
C18:2n6c	.345	.272	.934**	.000	.679*	.015	.271	.420	.642*	.024	.642*	.024
C18:3n6	.421	.173	.989**	.000	.803**	.002	.442	.174	.775**	.003	.784**	.003
C18:3n3	-.903**	.000	-.466	.127	-.563	.057	-.929**	.000	-.796**	.002	-.805**	.002
C20:0	.447	.145	-.214	.504	-.222	.487	.549	.080	.236	.460	.180	.575
C20:1n9	-.124	.700	.671*	.017	.500	.098	-.215	.525	.195	.544	.209	.514
C20:2	.591*	.043	.706*	.010	.438	.154	.558	.074	.756**	.004	.717**	.009
C20:3n6	-.703*	.011	-.820**	.001	-.868**	.000	-.758**	.007	-.842**	.001	-.902**	.000
C20:4n6	-.569	.054	-.098	.761	-.089	.783	-.534	.091	-.410	.186	-.435	.157

Lipid-soluble compounds	DPPH scavenging activity		FRAP		NO-inhibition in pre-incubated cell culture model (at 25 μg/ml)		NO-inhibition in co-incubated cell culture model (at 25 μg/ml)		NO-inhibition in pre-incubated cell culture model (at 50 μg/ml)		NO-inhibition in co-incubated cell culture model (at 50 μg/ml)	
	Pearson Correlation	Sig. (2-tailed)	Pearson Correlation	Sig. (2-tailed)	Pearson Correlation	Sig. (2-tailed)	Pearson Correlation	Sig. (2-tailed)	Pearson Correlation	Sig. (2-tailed)	Pearson Correlation	Sig. (2-tailed)
C20:3n3	-.525	.080	.178	.580	.154	.633	-.486	.129	-.242	.448	-.222	.488
C22:0	-.527	.078	-.814**	.001	-.837**	.001	-.422	.196	-.562	.057	-.689*	.013
C20:5n3	-.509	.091	.220	.492	.136	.673	-.526	.096	-.217	.498	-.222	.487
C22:1n9	-.076	.814	-.537	.072	-.705*	.011	-.057	.867	-.201	.531	-.331	.294
C24:0	-.005	.989	-.122	.707	-.330	.295	.114	.739	.162	.614	-.048	.882
C22:6	.716**	.009	.487	.108	.372	.234	.732*	.010	.767**	.004	.713**	.009
Unidentified	-.393	.207	-.621*	.031	-.416	.178	-.391	.235	-.520	.083	-.577*	.049
PUFA	-.674*	.016	-.030	.927	-.116	.718	-.673*	.023	-.440	.152	-.455	.137
SFA	.719**	.008	.218	.497	.232	.467	.728*	.011	.550	.064	.588*	.044
MUFA	.661*	.019	.637*	.026	.463	.130	.631*	.037	.698*	.012	.738**	.006
PUFA/SFA	-.752**	.005	-.192	.551	-.249	.434	-.759**	.007	-.564	.056	-.589*	.044
ARA/EPA	.485	.110	.960**	.000	.753**	.005	.482	.133	.761**	.004	.815**	.001

Note: *. Correlation is significant at the 0.05 level (2-tailed); **. Correlation is significant at the 0.01 level (2-tailed).
